# Diamond on Kurepa trees

**DOI:** 10.1007/s00605-026-02176-4

**Published:** 2026-03-28

**Authors:** Ziemowit Kostana, Assaf Rinot, Saharon Shelah

**Affiliations:** 1 Institute of Mathematics of the Czech Academy of Sciences, Prague, Czech Republic; 2 Department of Mathematics, Bar-Ilan University, Ramat-Gan, 5290002 Israel; 3 Einstein Institute of Mathematics, The Hebrew University of Jerusalem, Jerusalem, Israel; 4 Department of Mathematics, Rutgers University, Piscataway, U.S.A.

**Keywords:** Weak Kurepa tree, Diamond, Forcing with side conditions, Kurepa hypothesis, Primary 03E35

## Abstract

We introduce a new weak variation of diamond that is meant to guess only the branches of a Kurepa tree. We demonstrate that this variation is considerably weaker than diamond by proving it is compatible with Martin’s axiom. We then prove that this principle is nontrivial by showing it may consistently fail.

## Introduction

Kurepa [25] initiated the systematic study of uncountable trees through his attempt to solve Souslin’s problem [38] that asks whether the real line is the unique dense complete linear order with no endpoints satisfying that every pairwise disjoint collection of open intervals is countable. Kurepa showed that a counterexample is equivalent to the existence of what he called a *Souslin tree*. After sharing his findings with Aronszajn, the latter was able to construct a poor man’s version of a Souslin tree that Kurepa then named an *Aronszajn tree*. A third type of an uncountable tree is now known as a *Kurepa tree*. These trees and their higher analogs were central in the development of set theory, where quite a few key concepts and techniques in forcing theory and large cardinals were discovered through solving problems concerning these objects. A milestone work here is Jensen’s study [20] of the fine structure of Gödel’s inner model $\textsf{L}$ [15]. First, Jensen proved that a Souslin tree exists in $\textsf{L}$ [21]. Soon after, Solovay proved that a Kurepa tree exists in $\textsf{L}$ and Silver [36] extended it to outer universes under an optimal anti-large-cardinal assumption. Jensen (with a touch by Kunen) formulated the axiom $\diamondsuit$ as a *guessing principle* that holds in $\textsf{L}$ but is sufficient for the construction of a Souslin tree in any universe of set theory [20], and then Jensen, Kunen and Silver devised the stronger variation $\diamondsuit ^+$ as an axiom sufficient for the construction of a Kurepa tree [22] .

Uncountable trees and diamond principles found many applications in infinite Ramsey theory, topology, measure theory and algebra. To give just one example, we mention that in [32], the third author proved that $\diamondsuit ^+$ is sufficient to imply that all Whitehead groups of size $\aleph _1$ are free. And there are in addition some indirect applications, e.g., the Higman-Stone construction of an uncountable inverse system of rings whose limit is empty [17] which involves the rediscovery of an Aronszajn tree.

By now, a good understanding of diamond principles has been reached. For instance, the connection to other postulates such as the continuum hypothesis and forcing axioms has been determined [5, 10, 11, 14, 35, 37, 39] and pump-up lemmas were discovered showing that weaker diamonds can be made stronger by various combinatorial maneuvers [8, 9, 24, 26, 34].

Motivated by the current state of this understanding and by the fact that $\diamondsuit ^+$ is actually equivalent to the existence of a particular sort of a Kurepa tree [24, Exercise VI.9], in this paper we revisit the very basics by proposing a generalization of diamond that becomes most interesting when tested against Kurepa trees and prove that all the familiar features of diamonds break down in the new context. Unlike the usual diamond, our new diamond does not imply the continuum hypothesis and it is compatible with forcing axioms. Furthermore, it is indestructible under various notions of forcing and can be made indestructible under all *proper* forcings. Three additional features that the classical diamond possesses and our new diamond lacks are: guessing uncountably often is equivalent to guessing stationarily often, guessing correctly modulo a countable error is equivalent to guessing correctly with no errors, and the feature that diamond cannot be added by a ccc forcing.

Next we make things precise. For this, let us first recall the definition of the trees under discussion.

### Definition 1.1

A *binary tree of height* $\omega _1$ is a set *T* satisfying the following three requirements:

, i.e., *T* consists of binary sequences of countable length;
- *T* is downward-closed, i.e., for all $t\in T$ and $\alpha <{{\,\textrm{dom}\,}}(t)$, $t\mathbin \upharpoonright \alpha \in T$;
- for every $\alpha <\omega _1$, the $\alpha ^{\text {th}}$ level of *T*, $T_\alpha :=\{ t\in T\mid {{\,\textrm{dom}\,}}(t)=\alpha \}$, is nonempty.

We denote by  the collection of all *cofinal branches* through *T*.

We now arrive at the main definition of this paper.

### Definition 1.2

For a binary tree *T* of height $\omega _1$, the axiom $\diamondsuit (T)$ asserts the existence of a sequence $\langle t_\alpha \mid \alpha <\omega _1\rangle$ such that:

- for every $\alpha <\omega _1$, $t_\alpha$ is a function from $\alpha$ to 2;
- for every $f\in \mathcal {B}(T)$, the set $\{\alpha <\omega _1\mid f\mathbin \upharpoonright \alpha =t_\alpha \}$ is stationary.^1^

Modulo the identification of sets with their characteristic functions, the classical $\diamondsuit$ is by definition equivalent to , where  is the complete binary tree of height $\omega _1$. In particular, $\diamondsuit$ implies $\diamondsuit (T)$ for every *T*.

A decomposition theorem of Ulam [43] easily implies that $\diamondsuit (T)$ holds for every *T* such that $|\mathcal {B}(T)|\le \aleph _1$. In addition, if $\diamondsuit (T)$ holds then so does $\diamondsuit (T')$ for some tree $T'$ of size $\aleph _1$ with $\mathcal {B}(T')=\mathcal {B}(T)$. When put together, this shows that the study of $\diamondsuit (T)$ should focus on those *T* of size $\aleph _1$ such that $|\mathcal {B}(T)|>\aleph _1$. This is a well-known class of trees. Indeed, it is the first class of the following definition.

### Definition 1.3

A binary tree *T* of height $\omega _1$ is:

1. *weak Kurepa* iff $|T|=\aleph _1<|\mathcal {B}(T)|$;^2^
2. *Kurepa* iff $|T_\alpha |<\aleph _1$ for all $\alpha <\omega _1$ and $|\mathcal {B}(T)|>\aleph _1$;
3. *Aronszajn* iff $|T_\alpha |<\aleph _1$ for all $\alpha <\omega _1$ and $\mathcal {B}(T)=\emptyset$;
4. *Souslin* iff it is Aronszajn and for every uncountable $X\subseteq T$ there are $x,y\in X$ with $x\subsetneq y$.

Note that by Cantor’s theorem,  happens to be a weak Kurepa tree iff the continuum hypothesis holds. Also note that it follows from the previous discussion and the trivial fact that every Kurepa tree is a weak Kurepa tree that the failure of diamond over a Kurepa tree would constitute the ultimate failure of our new principle, hence also of the classical one.

### Main results

We will establish that $\diamondsuit \implies \clubsuit \implies \clubsuit _w\implies \diamondsuit (T)$ for every *T* of size $\aleph _1$, and show that our new principle is considerably weaker than the other ones. Moreover, it will be shown that Martin’s axiom is compatible with $\diamondsuit (T)$ holding over a binary Kurepa tree *T*. This is a corollary to any of the following two results:^3^

#### Theorem A

If *T* and *S* are two binary Kurepa trees and $|\mathcal {B}(T)|<|\mathcal {B}(S)|$, then $\diamondsuit (T)$ holds.

#### Theorem B

It is consistent that there exists a binary Kurepa tree *T* such that $\diamondsuit (T)$ holds and cannot be killed by a proper forcing.^4^

There are additional results that we already hinted upon earlier on, but the main result of this paper is the finding that this very weak variation of diamond is nevertheless nontrivial:

#### Theorem C

It is consistent that $\diamondsuit (T)$ fails for some binary Kurepa tree *T*. Furthermore, such a tree *T* can be chosen to be either rigid or homogeneous. Furthermore, the failure of $\diamondsuit (T)$ can be arranged together with $\diamondsuit (T')$ holding for some binary Kurepa tree $T'$ having the same number of branches as that of *T*.

The model witnessing the preceding is obtained by a countable support iteration of proper notions of forcing for adding a branch that evades a potential diamond sequence using models as side conditions. Curiously, the said Kurepa tree *T* will start its life in the ground model as a particular Aronszajn tree obtained from $\diamondsuit ^+$.

### Organization of this paper

In Sect. 2, we provide some preliminaries on trees, justify our focus on Kurepa trees that are binary and compare the principle $\diamondsuit (T)$ with other standard set-theoretic hypotheses. The proof of Theorem A will be found there.

In Sect. 3, we study the existence of Kurepa trees *T* such that $\diamondsuit (T)$ holds in an indestructible way. The proof of Theorem B will be found there.

In Sect. 4, we present a notion of forcing $\mathbb {Q}(T,\,\vec {t})$ to add a branch through an $\aleph _1$-tree *T* that evades a given potential diamond sequence $\vec {t}=\langle t_\alpha \mid \alpha <\omega _1\rangle$. We give a sufficient condition for $\mathbb {Q}(T,\,\vec {t})$ to be proper, and then turn to iterate it. The proof of Theorem C will be found there.

## Warm up

### Forcing background

We assume the reader is comfortable with the method of forcing [24], and is familiar with proper forcing [1, 33], its characterization in terms of preservation of generalized stationary sets, and the fact that this class is closed under 2-step iterations as well as under countable support iterations.

### Abstract, Hausdorff and binary trees

A *tree* is a partially ordered set $\textbf{T}=(T,<_T)$ such that, for every $x\in T$, the cone $x_\downarrow :=\{y\in T\mid y<_T x\}$ is well-ordered by $<_T$; its order type is denoted by ${{\,\textrm{ht}\,}}(x)$. For any ordinal $\alpha$, the $\alpha ^{\text {th}}$-level of the tree is the collection $T_\alpha :=\{ x\in T\mid {{\,\textrm{ht}\,}}(x)=\alpha \}$. The *height* of the tree is the first ordinal $\alpha$ for which $T_\alpha =\emptyset$. The tree is *normal* iff for every $t\in T$ and every ordinal $\alpha$ in-between ${{\,\textrm{ht}\,}}(x)$ and the height of the tree, there exists some $y\in T_\alpha$ with $x<_Ty$. The tree is *Hausdorff* iff for every limit ordinal $\alpha$ and all $x,y\in T_\alpha$, if $x_\downarrow =y_\downarrow$, then $x=y$. In particular, a (nonempty) Hausdorff tree has a unique root.

An $\aleph _1$-*tree* is a tree $\textbf{T}$ of height $\omega _1$ all of whose levels are countable. A *Kurepa tree* (resp. *Aronszajn tree*) is an $\aleph _1$-tree $\textbf{T}$ satisfying that the set $\mathcal {B}(\textbf{T})$ of all uncountable maximal chains in $\textbf{T}$ has size $\ge \aleph _2$ (resp. is empty). Definition 1.2 generalizes to abstract trees of height $\omega _1$ following Footnote 1 and by interpreting $f\mathbin \upharpoonright \alpha$ (for $f\in \mathcal {B}(\textbf{T})$ and $\alpha <\omega _1$) as the unique element of $T_\alpha$ that belongs to *f*.

Hereafter, whenever we talk about a *binary* $\aleph _1$-*tree*, we mean a set *T* as in Definition 1.1 such that $T_\alpha$ is countable for every $\alpha <\omega _1$, and we shall freely identify it with the Hausdorff $\aleph _1$-tree $\textbf{T}:=(T,{{\subsetneq }})$.

#### Lemma 2.1

For every Hausdorff $\aleph _1$-tree $\textbf{T}=(T,<_T)$, there exists a binary $\aleph _1$-tree *S* that is club-isomorphic to $\textbf{T}$, i.e., for some club $D\subseteq \omega _1$, $(\bigcup _{\alpha \in D}T_\alpha ,<_T)$ and $(\bigcup _{\alpha \in D}S_\alpha ,{\subsetneq })$ are order-isomorphic.

In particular, if $\textbf{T}$ is Kurepa, then *S* is Kurepa and $\diamondsuit (S)$ iff $\diamondsuit (\textbf{T})$.

#### Proof

Let $\textbf{T}=(T,<_T)$ be a given Hausdorff $\aleph _1$-tree. For every $\alpha <\omega _1$, fix an injection $\varphi _\alpha :T_{\alpha +1}\rightarrow \omega$. Also fix an injective sequence $\langle r_m\mid m<\omega \rangle$ of functions from $\omega$ to 2. We shall define an injection  satisfying the following two requirements:

1. for every $$t\in T$$, $${{\,\textrm{dom}\,}}(\psi (t))=\omega \cdot {{\,\textrm{ht}\,}}(t)$$;
2. for all $t',t\in T$, $t'<_T t$ iff $\psi (t')\subsetneq \psi (t)$.

The definition of $\psi$ is by recursion on the heights of the nodes in $\textbf{T}$. By Clause (1) we are obliged to send the unique root of $\textbf{T}$ to $\emptyset$ and by Clauses (1) and (2), for every $\alpha \in {{\,\textrm{acc}\,}}(\omega _1)$ and every $t\in T_\alpha$,^5^ we are obliged to set $\psi (t):=\bigcup \{\psi (t')\mid t'<_T t\}$.^6^ Thus, the only freedom we have is at nodes of successor levels. Here, for every $\alpha <\omega _1$ such that $\psi \mathbin \upharpoonright T_\alpha$ has already been defined, and for every $t\in T_{\alpha +1}$, let $t^-$ denote the immediate predecessor of *t*, and setFinally, consider $S:=\{ s\mathbin \upharpoonright \alpha \mid s\in {{\,\textrm{Im}\,}}(\psi ),~\alpha \le {{\,\textrm{dom}\,}}(s)\}$. It is clear that *S* is a downward-closed subfamily of . Thus, to see that *S* is a binary $\aleph _1$-tree, it suffices to prove that all of its levels are countable. However, by Clauses (1) and (2), more is true, namely, for every $\alpha <\omega _1$,

$$\begin{aligned} S_\alpha =\{ \psi (t)\mathbin \upharpoonright \alpha \mid t\in T_\alpha \}. \end{aligned}$$

Consider the club $D:=\{\alpha <\omega _1\mid \omega \cdot \alpha =\alpha \}$. Evidently, $\psi$ witnesses that $(\bigcup _{\alpha \in D}T_\alpha ,<_T)$ and $(\bigcup _{\alpha \in D}S_\alpha ,{\subsetneq })$ are order-isomorphic. $\square$

Any tree $\textbf{T}=(T,<_T)$ can be cofinally-embedded in a Hausdorff tree of the form $(S,{\subsetneq })$ by letting *S* be the downward closure of the collection of all $s:\alpha \rightarrow T$ that are order-preserving maps from *a successor* ordinal $(\alpha ,{\in })$ onto some downward-closed subset of $(T,<_T)$. The map that sends each $t\in T$ to the unique $s\in S$ with $\max ({{\,\textrm{Im}\,}}(s),<_T)=t$ embeds $\textbf{T}$ to the successor levels of *S*. In case that $\textbf{T}$ is non-Hausdorff, there is no better embedding. Since non-Hausdorff trees lack genuine limit levels and since our definition of diamond on trees has to do with stationary sets, the study here will be focused on the theory of diamond on Hausdorff Kurepa trees. By Lemma 2.1, then, we may moreover focus on binary Kurepa trees.

### Diamonds and clubs

As said before, $\diamondsuit \implies \clubsuit \implies \clubsuit _w\implies \diamondsuit (T)$ for those *T* of size $\aleph _1$. The first two implications are well-known. We now give the details of the last one.

#### Proposition 2.2

If $\clubsuit _w$ holds, then so does $\diamondsuit (T)$ for every binary tree *T* of height $\omega _1$ and size $\aleph _1$.

#### Proof

Suppose that $\clubsuit _w$ holds. By [12, p. 61], this means that we may fix a sequence $\langle B_\alpha \mid \alpha \in {{\,\textrm{acc}\,}}(\omega _1)\rangle$ such that each $B_\alpha$ is a cofinal subset of $\alpha$ of order-type $\omega$, and, for every uncountable $B\subseteq \omega _1$, the following set is stationary:

$$\begin{aligned} G(B):=\{\alpha \in {{\,\textrm{acc}\,}}(\omega _1)\mid B_\alpha \setminus B\text { is finite}\}. \end{aligned}$$

Now, suppose *T* is a binary tree of height $\omega _1$ and size $\aleph _1$. Fix a bijection $\pi :\omega _1\leftrightarrow T$. For every $\alpha \in {{\,\textrm{acc}\,}}(\omega _1)$, if there are $t_\alpha \in T_\alpha$ and a finite $F_\alpha \subseteq B_\alpha$ such that

$$\begin{aligned} t_\alpha =\bigcup \{\pi (\beta )\mid \beta \in B_\alpha \setminus F_\alpha \}, \end{aligned}$$

then we keep this unique $t_\alpha$; otherwise (including the case $\alpha \notin {{\,\textrm{acc}\,}}(\omega _1)$), we let $t_\alpha$ be an arbitrary choice of an element of $T_\alpha$. To see that $\langle t_\alpha \mid \alpha <\omega _1\rangle$ witnesses $\diamondsuit (T)$, let $f\in \mathcal {B}(T)$. Consider the following club:

$$\begin{aligned} D:=\{\delta \in {{\,\textrm{acc}\,}}(\omega _1) \mid \forall \gamma<\delta \,[\pi ^{-1}(f\mathbin \upharpoonright \gamma )<\delta ]\}. \end{aligned}$$

Note that for every $\delta \in D$, $\delta \le \pi ^{-1}(f\mathbin \upharpoonright \delta )<\min (D\setminus (\delta +1))$. In particular, the following set is uncountable:

$$\begin{aligned} B:=\{ \pi ^{-1}(f\mathbin \upharpoonright \delta )\mid \delta \in D\}. \end{aligned}$$

We claim that for every $\alpha \in G(B)$, it is the case that $f\mathbin \upharpoonright \alpha =t_\alpha$. Indeed, the set $F_\alpha :=B_\alpha \setminus B$ is finite, and for every $\beta \in B_\alpha \setminus F_\alpha$, there exists a unique $\delta \in D$ such that $\delta \le \beta =\pi ^{-1}(f\mathbin \upharpoonright \delta )<\min (D\setminus (\delta +1))$. So, since $\sup (B_\alpha \setminus F_\alpha )=\alpha$, it is the case that

$$\begin{aligned} t_\alpha =\bigcup \{\pi (\beta )\mid \beta \in B_\alpha \setminus F_\alpha \}=f\mathbin \upharpoonright \alpha , \end{aligned}$$

as sought. $$\square $$

Since the classical $\diamondsuit$ coincides with , the preceding yields the implication $\clubsuit \wedge \textsf {CH }\implies \diamondsuit$. Using [12, Lemma 3.7], it also implies that $\diamondsuit (T)$ is compatible with $\lnot \textsf {CH }$. An alternative reasoning goes through the next proposition. Namely, add $\aleph _2$ many Cohen reals to a model of $\diamondsuit ^+$.

#### Proposition 2.3

Suppose that $\textbf{T}$ is a Kurepa tree such that $\diamondsuit (\textbf{T})$ holds, and that $\mathbb {P}$ is a notion of forcing. If $\mathbb {P}$ is $\sigma$-closed or if $\mathbb {P}^2$ satisfies the ccc, then $\diamondsuit (\textbf{T})$ remains to hold in $V^{\mathbb {P}}$.

#### Proof

By [36], a $\sigma$-closed forcing does not add new branches to $\aleph _1$-trees. By [44, Lemma 2.2], a forcing notion whose square satisfies the ccc does not add new branches to $\aleph _1$-trees. In addition, stationary sets are preserved by $\sigma$-closed notions of forcing and by ccc notions of forcing. So, in both cases, the ground model witness to $\diamondsuit (\textbf{T})$ will survive as a witness in $V^{\mathbb {P}}$. $\square$

Another simple argument shows that an instance of the parameterized diamond principle from [29] is enough.

#### Proposition 2.4

If $\Phi (\omega ,=)$ holds, then so does $\diamondsuit (T)$ for every binary Kurepa tree *T*.

#### Proof

Suppose that $\Phi (\omega ,=)$ holds. This means that for every function , there exists a function $g:\omega _1\rightarrow \omega$ such that, for every function $f:\omega _1\rightarrow 2$, the following set is stationary:

$$\begin{aligned} G(f):=\{ \alpha <\omega _1\mid F(f\mathbin \upharpoonright \alpha )=g(\alpha )\}. \end{aligned}$$

Now, suppose *T* is a binary Kurepa tree. For every $\alpha <\omega _1$, fix an enumeration $\langle t^n_\alpha \mid n<\omega \rangle$ of $T_\alpha$. Fix a function  such that for all $\alpha <\omega _1$ and $t\in T_\alpha$,

$$\begin{aligned} F(t)=\min \{n<\omega \mid t=t_\alpha ^n\}, \end{aligned}$$

and then let $g:\omega _1\rightarrow \omega$ be the corresponding function given by $\Phi (\omega ,{=})$. A moment’s reflection makes it clear that the transversal $\langle t_\alpha ^{g(\alpha )} \mid \alpha < \omega _1\rangle$ witnesses $\diamondsuit (T)$. $\square$

Recall that a *Souslin tree* is an Aronszajn tree with no uncountable antichains. Every Souslin tree contains a Souslin subtree that is normal. As established in [29], forcing with such trees gives an instance of $\Phi (\omega ,=)$. A variant of that argument gives the following.

#### Proposition 2.5

Forcing with a normal Souslin tree adds a $\diamondsuit (T)$-sequence for every ground model binary $\aleph _1$-tree *T*.

#### Proof

Working in *V*, suppose that $\textbf{S}=(S,{<_S})$ is a normal Souslin tree, and that *T* is a binary $\aleph _1$-tree. As $\textbf{S}$ is normal and $\mathcal {B}(\textbf{S})=\emptyset$, for each $s\in S$, we may let $\langle s^n \mid n<\omega \rangle$ be a bijective enumeration of some infinite maximal antichain above *s*. For every $\alpha <\omega _1$, fix an enumeration $\langle t^n_\alpha \mid n<\omega \rangle$ of $T_\alpha$. Given a generic *G* for $\textbf{S}^*:=(S,{>_S})$, for every $\alpha <\omega _1$, denote by $s_\alpha$ the unique element of $S_\alpha$ that belongs to *G*, and then let $g(\alpha )$ denote the unique integer $n<\omega$ such that $(s_\alpha )^n$ belongs to *G*. We claim that the transversal $\langle t_\alpha ^{g(\alpha )} \mid \alpha < \omega _1\rangle$ witnesses $\diamondsuit (T)$.

To see this, back in *V*, fix a condition $s \in S$, a name $\dot{f}$ for a cofinal branch through *T*, and a club $C\subseteq \omega _1$; we shall find an $\alpha \in C$ and an extension of *s* forcing that $\dot{f}\mathbin \upharpoonright \alpha$ coincides with $t_\alpha ^{\dot{g}(\alpha )}$. Note that since $\textbf{S}^*$ is ccc, we can indeed restrict our attention to ground model clubs.

Fix a countable $M\prec \operatorname {H}_{\omega _2}$, containing $\{\dot{f},s,\textbf{S}^*,T,C\}$. Write $\alpha :=M\cap \omega _1$, and note that $\alpha \in C$, since $C\in M$. As $\textbf{S}$ is normal, fix $s' \in S_\alpha$ extending *s*. By a folklore fact, $s'$ is an $\textbf{S}^*$-generic branch over *M*, so in particular it determines $\dot{f}\mathbin \upharpoonright \alpha$, and therefore there is an integer *n* such that

$$\begin{aligned} s' \Vdash \dot{f}\mathbin \upharpoonright \alpha = t_\alpha ^n. \end{aligned}$$

Set $s'':=(s')^n$, so that $s''\Vdash \dot{g}(\alpha )=n$. Then $s''\mathrel {>_S}s'\mathrel {>_S}s$, and

$$\begin{aligned} s'' \Vdash \dot{f}\mathbin \upharpoonright \alpha = t_\alpha ^{\dot{g}(\alpha )}, \end{aligned}$$

as sought. $$\square $$

#### Corollary 2.6

Suppose *T* is a normal binary Souslin tree. If *T* is almost-Kurepa, then in some ccc forcing extension, *T* is a binary Kurepa tree and $\diamondsuit (T)$ holds.

#### Proof

By definition, *T* is *almost-Kurepa* iff in the forcing extension by $\mathbb {P}:=(T,{\supseteq })$, *T* is a Kurepa tree. By Proposition 2.5, $\diamondsuit (T)$ will hold in $V^{\mathbb {P}}$. $\square$

### Weak variations

Devlin [9, §2] showed that if there exists a sequence  such that, for every function $f:\omega _1\rightarrow 2$, there is an infinite ordinal $\alpha <\omega _1$ with $f\mathbin \upharpoonright \alpha =t_\alpha$, then $\diamondsuit$ holds. When combined with Theorem C, the next proposition shows that this does not generalize to our context.

#### Proposition 2.7

Every $\aleph _1$-tree $\textbf{T}$ admits a transversal $\langle t_\alpha \mid \alpha <\omega _1\rangle$ such that $G(f):=\{ \alpha <\omega _1\mid f\mathbin \upharpoonright \alpha =t_\alpha \}$ is uncountable for every $f\in \mathcal {B}(\textbf{T})$.

#### Proof

Suppose that $\textbf{T}=(T,<_T)$ is a an $\aleph _1$-tree. Recall that for all $f\in \mathcal {B}(\textbf{T})$ and $\alpha <\omega _1$, $f\mathbin \upharpoonright \alpha$ denotes the unique element of $T_\alpha$ that belongs to *f*. Likewise, for every $t\in T$, and $\alpha \le {{\,\textrm{ht}\,}}(t)$, we denote by $t\mathbin \upharpoonright \alpha$ the unique element of $T_\alpha$ that is $<_T$-comparable with *t*.

Now, for every limit ordinal $\beta <\omega _1$, fix a surjection $\phi _\beta :\omega \rightarrow T_{\beta +\omega }$, and then for every $n<\omega$, let $t_{\beta +n}:=\phi _\beta (n)\mathbin \upharpoonright (\beta +n)$. To see that $\langle t_\alpha \mid \alpha <\omega _1\rangle$ is as sought, let $f\in \mathcal {B}(T)$. For every limit $\beta <\omega _1$, as $f\mathbin \upharpoonright (\beta +\omega )$ is in *T*, we may find some $n<\omega$ such that $\phi _\beta (n)=f\mathbin \upharpoonright (\beta +\omega )$. Consequently,

$$\begin{aligned} f\mathbin \upharpoonright (\beta +n)=\phi _\beta (n)\mathbin \upharpoonright (\beta +n)=t_{\beta +n}. \end{aligned}$$

It follows that for some stationary $B\subseteq \omega _1$ and some $n'<\omega$, $G(f)\supseteq \{ \beta +n'\mid \beta \in B\}$. In particular, *G*(*f*) is uncountable. $\square$

In [24, Theorem II.7.14], Kunen proved that $\diamondsuit ^-$ is equivalent to $\diamondsuit$. This means that if there exists a sequence  such that, for every function $f:\omega _1\rightarrow 2$, the set $\{\alpha <\omega _1\mid f\mathbin \upharpoonright \alpha \in T_\alpha \}$ is stationary, then there exists a sequence  such that, for every function $f:\omega _1\rightarrow 2$, the set $\{\alpha <\omega _1\mid f\mathbin \upharpoonright \alpha = t_\alpha \}$ is stationary.

In contrast, if $\textbf{T}$ is an $\aleph _1$-tree, then for every $\alpha <\omega _1$, $T_\alpha$ is a countable subset of  and for every $f\in \mathcal {B}(\textbf{T})$, the set $\{\alpha <\omega _1\mid f\mathbin \upharpoonright \alpha \in T_\alpha \}$ is the whole of $\omega _1$. By Theorem C, this shows that in our context, guessing correctly modulo a countable error is not equivalent to guessing correctly.

### Additional ways to get diamond

Kunen proved that $\diamondsuit$ cannot be introduced by a ccc forcing [24, Exercise VII.H9], whereas Proposition 2.5 demonstrates that this is not the case with $\diamondsuit (T)$. Another small ccc forcing that adds a diamond sequence for every ground model Kurepa tree is Cohen’s forcing ${{\,\textrm{Add}\,}}(\omega ,1)$. The core of this fact can be restated combinatorially, as follows.

#### Proposition 2.8

Suppose that $\textbf{T}$ is a Kurepa tree. If ${{\,\textrm{cov}\,}}(\mathcal {M})>|\mathcal {B}(\textbf{T})|$,^7^ then $\diamondsuit (\textbf{T})$ holds.

#### Proof

For every $\alpha <\omega _1$, fix an enumeration $\langle t^n_\alpha \mid n<\omega \rangle$ of $T_\alpha$. Fix a partition $\langle S_i\mid i<\omega \rangle$ of $\omega _1$ into stationary sets, and let $\pi :\omega _1\rightarrow \omega$ be such that $\pi [S_i]=\{i\}$ for every $i<\omega$. For each $f\in \mathcal {B}(\textbf{T})$, define a map $r_f:\omega \rightarrow \omega$ via:

$$\begin{aligned} r_f(i):=\min \{n<\omega \mid \{\alpha \in S_i\mid f\mathbin \upharpoonright \alpha =t_\alpha ^n\}\text { is stationary}\}. \end{aligned}$$

Finally, assuming ${{\,\textrm{cov}\,}}(\mathcal {M})>|\mathcal {B}(\textbf{T})|$, by [3, Lemma 2.4.2], we may fix a function $g:\omega \rightarrow \omega$ such that $r_f\cap g\ne \emptyset$ for every $f\in \mathcal {B}(\textbf{T})$. Then the transversal $\langle t_\alpha ^{g(\pi (\alpha ))} \mid \alpha < \omega _1\rangle$ witnesses $\diamondsuit (\textbf{T})$. $\square$

#### Remark 2.9

$\diamondsuit ^+$ implies that $\diamondsuit (\textbf{T})$ holds for some Kurepa tree $\textbf{T}$ with ${{\,\textrm{cov}\,}}(\mathcal {M})<|\mathcal {B}(\textbf{T})|$. In Corollary 3.5 below, we get a model in which $\diamondsuit (\textbf{T})$ holds for some Kurepa tree $\textbf{T}$ with ${{\,\textrm{cov}\,}}(\mathcal {M})=|\mathcal {B}(\textbf{T})|$.

#### Remark 2.10

A proof similar to that of Proposition 2.8 shows that $\diamondsuit (\textbf{T})$ holds for every Kurepa tree $\textbf{T}$ with $\mathfrak d_{\omega _1}>|\mathcal {B}(\textbf{T})|$.

Our next task is proving Theorem A. For this, let us recall the following definition.

#### Definition 2.11

A tree $\textbf{T}=(T,<_T)$ of height $\omega _1$ is:

- a *weak Kurepa tree* iff $|T|=\aleph _1<|\mathcal {B}(\textbf{T})|$;
- a *Jech–Kunen tree* iff $|T|=\aleph _1<|\mathcal {B}(\textbf{T})|<2^{\aleph _1}$;
- *thick* iff $$|\mathcal {B}(\textbf{T})|=2^{\aleph _1}$$.

As mentioned earlier, $\textsf {CH }$ implies that  is a thick weak Kurepa tree.

#### Proposition 2.12

Suppose that $\textbf{T}$ is a Kurepa tree, $\textbf{S}$ is a weak Kurepa tree, and $|\mathcal {B}(\textbf{T})|<|\mathcal {B}(\textbf{S})|$. Then $\diamondsuit (\textbf{T})$ holds.

#### Proof

By [18, Corollary 7.18], if $\kappa$ is a regular uncountable cardinal and there is a Kurepa tree with (at least) $\kappa$-many branches, then ${{\,\mathrm{\textsf{onto}}\,}}(\{\aleph _1\},J^{{{\,\textrm{bd}\,}}}[\kappa ],\aleph _0)$ holds. The same proof works equally well against weak Kurepa trees. Therefore, $\kappa :=|\mathcal {B}(\textbf{T})|^+$ is a regular uncountable cardinal such that ${{\,\mathrm{\textsf{onto}}\,}}(\{\aleph _1\},J^{{{\,\textrm{bd}\,}}}[\kappa ],\aleph _0)$ holds. This means that we may fix a map $c:\omega _1\times \kappa \rightarrow \omega$ such that for every $B\in [\kappa ]^\kappa$, there exists $i<\omega _1$ such that $c[\{i\}\times B]=\omega$. For each $\beta <\kappa$, define $g_\beta :\omega _1\rightarrow \omega$ via $g_\beta (i):=c(i,\beta )$. It is easy to check that for every , there exists some $\beta <\kappa$ such that $r\cap g_\beta \ne \emptyset$ for every $r\in \mathcal {R}$. From this point on, the proof continues the same way as that of Proposition 2.8, where the only change is that we fix a partition $\langle S_i\mid i<\omega _1\rangle$ into $\aleph _1$-many stationary sets and so each of the $r_f$’s is now a function from $\omega _1$ to $\omega$. $\square$

#### Corollary 2.13

$\textsf {CH }$ implies $\diamondsuit (\textbf{T})$ for every non-thick Kurepa tree $\textbf{T}$. $\square$

## Indestructible diamonds

We would like to show that if the universe is close to being constructible, then there exists a Kurepa tree on which $\diamondsuit$ holds. The construction of such a tree relies on the concept of a *sealed* Kurepa tree:

### Definition 3.1

(Hayut-Müller, [16]) A Kurepa tree $\textbf{T}$ is *sealed* if for every notion of forcing $\mathbb {P}$ that preserves both $\omega _1$ and $\omega _2$, $\mathbb {P}$ does not add a new branch through $\textbf{T}$.

### Fact 3.2

(Poór-Shelah, [30, §4]) If $\omega _1=\omega _1^{\textsf{L}[A]}$ and $\omega _2=\omega _2^{\textsf{L}[A]}$ for some $A \subseteq \omega _1$, then there exists a Kurepa tree $\textbf{T}$ such that $\mathcal {B}(\textbf{T}) \subseteq \textsf{L}[A]$.^8^

### Corollary 3.3

(Giron-Hayut, [13]) If $\omega _1=\omega _1^{\textsf{L}[A]}$ and $\omega _2=\omega _2^{\textsf{L}[A]}$ for some $A \subseteq \omega _1$, then there exists a sealed Kurepa tree. $\square$

### Corollary 3.4

Suppose that $V=\textsf{L}[A]$ for some $A\subseteq \omega _1$. Then there exists a binary Kurepa tree *T* such that $\diamondsuit (T)$ holds in any forcing extension preserving $\omega _1$, $\omega _2$, and the stationary subsets of $\omega _1$.

### Proof

Let $\textbf{T}$ be a tree as in Fact 3.2. It can be verified that $\textbf{T}$ is Hausdorff, but, regardless, as described right after Lemma 2.1, there is a Hausdorff tree $\textbf{S}$ and an order-preserving injection $\pi$ from $\textbf{T}$ to $\textbf{S}$ such that $\pi [T_\alpha ]=S_{\alpha +1}$ for every $\alpha <\omega _1$. In particular, $\pi$ induces a bijective correspondence between $\mathcal {B}(\textbf{T})$ and $\mathcal {B}(\textbf{S})$, meaning that we may as well assume that $\textbf{T}$ is Hausdorff. Next, as $A\subseteq \omega _1$, it is the case that $\diamondsuit$ holds in $\textsf{L}[A]$, so $\diamondsuit (\textbf{T})$ holds as well. Suppose $\mathbb {P}$ is a notion of forcing preserving $\omega _1$ and $\omega _2$. The construction of $\textbf{T}$ takes place inside $\textsf{L}[A]$, so

$$\begin{aligned} V^{\mathbb {P}} \models \mathcal {B}(\textbf{T}) \subseteq \textsf{L}[A]. \end{aligned}$$

As the definition of $\textsf{L}[A]$ is absolute, all the branches through $\textbf{T}$ from $V^{\mathbb {P}}$ are already in *V*. Now, if $\mathbb {P}$ also preserves stationary subsets of $\omega _1$, then the witness to $\diamondsuit (\textbf{T})$ in *V* will still witness $\diamondsuit (\textbf{T})$ in $V^{\mathbb {P}}$. Finally, by running the translation procedure of Lemma 2.1, we obtain a binary Kurepa tree with the same key features as the Hausdorff tree $\textbf{T}$. $\square$

### Corollary 3.5

Each of the following is compatible with $\diamondsuit (T)$ holding for some binary Kurepa tree *T*:

1. Martin’s axiom and $2^{\aleph _0}=\aleph _2$;
2. There are no Souslin trees (in particular, $\diamondsuit$ fails), and $\textsf {GCH }$ holds.

### Proof

Work in $\textsf{L}$. By Corollary 3.4, we may fix a binary Kurepa tree *T* such that $\diamondsuit (T)$ holds in any forcing extension preserving $\omega _1$, $\omega _2$, and the stationary subsets of $\omega _1$.

(1) Let $\mathbb {P}$ be some ccc notion of forcing such that in $\mathsf L^{\mathbb {P}}$, Martin’s axiom holds and $2^{\aleph _0}=\aleph _2$. As $\mathbb {P}$ preserves $\omega _1$, $\omega _2$, and the stationary subsets of $\omega _1$, $\diamondsuit (T)$ holds in $\mathsf L^{\mathbb {P}}$.

(2) As $\textsf {GCH }$, $\diamondsuit$ and $\square _{\aleph _1}$ all hold, we may let $\mathbb {P}$ be Jensen’s poset for forcing $\textsf {GCH }$ and the Souslin hypothesis (see [10]). $\mathbb {P}$ preserves $\omega _1$, $\omega _2$, and the stationary subsets of $\omega _1$, hence $\diamondsuit (T)$ holds in $\mathsf L^{\mathbb {P}}$. $\square$

A Kurepa tree that is sealed for all proper forcings can also be added by forcing, and in fact such a notion of forcing was already devised by D. H. Stewart in his 1966 Master’s thesis.

### Definition 3.6

(Stewart [40], see [19]) For a cardinal $\kappa >\aleph _1$, the forcing $\mathbb {S}_\kappa$ consists of all triples $(T_p,\epsilon _p,b_p)$ such that:

1. $$\epsilon _p\in {{\,\textrm{acc}\,}}(\omega _1)$$;
2. $T_p$ is a countable downward-closed subfamily of  such that $(T_p,{\subsetneq })$ is a normal tree of height $\epsilon _p+1$;
3. $$ b_p$$ is an injection from a countable subset of $$\kappa $$ to the top level of $$T_p$$,

and the ordering is given by $q \le p$ iff

- $$\epsilon _q\ge \epsilon _p$$;
- $$T_q\supseteq T_p$$ and $$T_q\mathbin \upharpoonright (\epsilon _p+1)=T_p$$;
- $${{\,\textrm{dom}\,}}(b_q)\supseteq {{\,\textrm{dom}\,}}(b_p)$$;
- for every $$\xi \in {{\,\textrm{dom}\,}}(b_p)$$, $$b_q(\xi )\supseteq b_p(\xi )$$.

Note that $\mathbb {S}_\kappa$ is $\sigma$-closed, and that, assuming $\textsf {CH }$, it has the $\aleph _2$-cc. The following establishes Theorem B.

### Proposition 3.7

Suppose $\kappa >\aleph _1$ is some cardinal. In the forcing extension by $\mathbb {S}_\kappa$, there exists a binary Kurepa tree *T* such that $\diamondsuit (T)$ holds and no proper forcing adds a new branch through *T*, let alone kills diamond over it.

### Proof

Suppose *G* is $\mathbb {S}_\kappa$-generic. Let $T:=\bigcup \{T_p\mid p\in G\}$ denote the generic binary tree added by $\mathbb {S}_\kappa$. For each $\xi <\kappa$, let

$$\begin{aligned} f_\xi :=\{ t\in T\mid \exists p\in G\,(\xi \in {{\,\textrm{dom}\,}}(b_p)\wedge t\subseteq b_p(\xi ))\}. \end{aligned}$$

A density argument shows that *T* is an $\aleph _1$-tree and that $\langle f_\xi \mid \xi <\kappa \rangle$ is an injective sequence of branches through it. By a theorem of Baumgartner, any $\sigma$-closed forcing that adds a new subset of $\omega _1$ forces $\diamondsuit$ to hold (see [31, Theorem 3.1]), hence $\diamondsuit (T)$ hold in *V*[*G*].

In [19, p. 10], Jech proved that $\{f_\xi \mid \xi < \kappa \}$ enumerates *all* cofinal branches through *T*. It is a folklore fact that this remains the case in any extension by a proper notion of forcing. Hints of this fact may be found in [41, Lemma 8.14] and [23, Proposition 37], but for completeness, we give the details here. Following [23, Definition 1], we consider the following set:

$$\begin{aligned} \mathcal {S}:=\{X \in [\kappa ]^{\aleph _0} \mid X\cap \omega _1 \in {{\,\textrm{acc}\,}}(\omega _1),\; T_{X\cap \omega _1}=\{f_\xi \mathbin \upharpoonright (X\cap \omega _1)\mid \xi \in X\}\}. \end{aligned}$$

### Claim 3.7.1

$$\mathcal {S}$$ is stationary.

### Proof

Back in *V*, pick a name $\dot{C}$ for a club in $[\kappa ]^{\aleph _0}$, and a condition *p*. Recursively construct a sequence $\langle (p_n,X_n)\mid n<\omega \rangle$ such that $(p_0,X_0):=(p,\omega )$ and, for every $n<\omega$:

- $$p_{n+1}\le p_n$$;
- $$\epsilon _{p_{n+1}}>\epsilon _{p_n}$$;
- $${{\,\textrm{dom}\,}}(b_{p_{n+1}})\supseteq X_n$$;
- every element of $T_{{p_n}}$ is extended by some element of ${{\,\textrm{Im}\,}}(b_{p_{n+1}})$;
- $$X_{n+1}\in [\kappa ]^{\aleph _0}$$ with $$X_{n+1}\supseteq X_n\cup {{\,\textrm{dom}\,}}(b_{p_n})\cup (\sup (X_n\cap \omega _1)+1)$$;
- $$p_{n+1}\Vdash X_{n+1}\in \dot{C}$$.

Set $X:=\bigcup _{n<\omega }X_n$ and note that $X\cap \omega _1\in {{\,\textrm{acc}\,}}(\omega _1)$. Define a condition *q* by letting:

1. $${{\,\textrm{dom}\,}}(b_q):=\bigcup _{n<\omega }X_n$$;
2. for all $n<\omega$ and $\xi \in X_n$, $b_q(\xi ):=\bigcup _{n<m<\omega }b_{p_m}(\xi )$;
3. $$\epsilon _q:=\sup _{n<\omega }\epsilon _{p_n}$$;
4. $$T_q:=\bigcup _{n<\omega }T_{p_n}\cup {{\,\textrm{Im}\,}}(b_{q})$$.

It is clear that $q\le p_n$ for all $n<\omega$, and hence $q\Vdash X\in \dot{C}$. In addition, Clause (4) implies that $q\Vdash X\in \dot{S}$. $\square$

Now, let $\mathbb {Q}$ be any proper forcing in *V*[*G*], and work in *V*[*G*][*H*], where *H* is $\mathbb {Q}$-generic. Towards a contradiction, suppose that *f* is a branch through *T* distinct from any of the $f_\xi$’s. As $\mathbb {Q}$ is proper, $\mathcal {S}$ remains stationary, so we may fix an elementary submodel $M\prec \operatorname {H}_\theta$ (for a large enough regular cardinal $\theta$) containing $\{T,f,\langle f_\xi \mid \xi <\kappa \rangle \}$ such that $X:=M\cap \kappa$ is in $\mathcal {S}$. By elementarity,

$$\begin{aligned} M\models \forall \xi <\kappa \,(f \ne f_\xi ). \end{aligned}$$

Denote $\alpha :=X\cap \omega _1$. As *f* is a branch through *T*, we get that $f\mathbin \upharpoonright \alpha \in T_\alpha$. As $X\in \mathcal {S}$, we may find a $\xi \in X$ such that $f\mathbin \upharpoonright \alpha =f_\xi \mathbin \upharpoonright \alpha$. But $\xi \in X = M\cap \kappa$, and then, by elementarity,

$$\begin{aligned} M \models f=f_\xi . \end{aligned}$$

This is a contradiction. $\square$

Despite the fact that the generic tree added by $\mathbb {S}_\kappa$ is sealed for proper forcings, we can still add new branches to it without collapsing cardinals or adding reals using the quotient forcing $\mathbb {S}_{\kappa ^+}/\mathbb {S}_{\kappa }$. More generally, consider the countable support iteration $(\langle \mathbb {P}_\xi \mid \xi \le \kappa \rangle ,\langle \dot{\mathbb {Q}}_\xi \mid \xi <\kappa \rangle )$, for an uncountable cardinal $\kappa$, where $\mathbb {Q}_0$ is the Jech partial order for adding a Souslin tree *T*, and, for every nonzero $\xi <\kappa$,

$$\begin{aligned} \mathbb {P}_\xi \ \Vdash \dot{\mathbb {Q}}_\xi = T. \end{aligned}$$

By Proposition 3.7 and the upcoming proposition, $\mathbb {P}_\kappa$ is proper (even $\sigma$-strategically closed) for any choice of $\kappa$, and yet the quotient forcings of the form $\mathbb {P}_\mu / \mathbb {P}_\lambda$ are not proper, whenever $\aleph _2 \le \lambda < \mu$.

### Proposition 3.8

$\mathbb {P}_\kappa$ has a dense subset that is isomorphic to $\mathbb {S}_\kappa$.

### Proof

Let *R* denote the collection of all *rectangular* conditions in $\mathbb {P}_\kappa$, i.e, the collection of all conditions *q* for which there exists some $\delta <\omega _1$ such that:

- *q*(0) is a tree of height $\delta +1$,
- for every $\xi \in \kappa \setminus \{0\}$, $q(\xi )$ is either trivial or a node at the $\delta ^{\text {th}}$-level of *q*(0),
- all maximal nodes of *q*(0) which are of the form $q(\xi )$, for $\xi \in \kappa \setminus \{0\}$, are pairwise distinct.

To see that *R* is isomorphic to $\mathbb {S}_\kappa$, note that we can map a condition $q \in R$ to a condition $\phi (q)$ in $\mathbb {S}_\kappa$, by keeping the tree coordinate, and using the function $b_{\phi (q)}$ to record all maximal nodes of *q*(0) that are of the form $q(\xi )$. More precisely, we put:

- $$T_{\phi (q)}(0):=q(0)$$,
- $$\epsilon _{\phi (q)}:={{\,\textrm{ht}\,}}(q(0))-1$$,
- $b_{\phi (q)}(\xi ):=q(\xi ),$ whenever $q(\xi )$ belongs to the top level of *q*(0).

This mapping is clearly order-preserving, with the image

$$\begin{aligned} \{p \in \mathbb {S}_\kappa \mid 0 \notin {{\,\textrm{dom}\,}}(b_p)\}, \end{aligned}$$

which is obviously isomorphic to $\mathbb {S}_\kappa$. Moreover, it is straightforward to define an inverse of $\phi$.

It remains to show that *R* is dense. Let *p* be an arbitrary condition in $\mathbb {P}_\kappa$. Fix a countable $M\prec \operatorname {H}_\theta$, for a sufficiently large regular cardinal $\theta$, containing all relevant objects. Let $\delta :=\omega _1 \cap M$, and let $G \subseteq P\cap M$ be a $\mathbb {P}_\kappa$-generic filter over *M*, containing *p*. For $\xi \in \kappa \cap M$, let us denote by $G(\xi )$ the projection of *G* to the coordinate $\xi$. Therefore $\bigcup G(0)$ is a tree of height $\delta$, with countable levels, and for each $\xi \in \kappa \cap M \setminus \{0\}$, $G(\xi )$ determines a cofinal branch $f_\xi$ through *G*(0), given by the formulaThe latter follows from the observation that for any $\epsilon <\delta$, the setis dense in $\mathbb {P}_\kappa$, and belongs to *M*.

Finally, we define a condition *q* by the following considerations:

- $$q(0):=G(0)\cup \{f_\xi \mid \xi \in M\cap \kappa \}$$,
- $$q(\xi ):=f_\xi $$ for every $$\xi \in M\cap \kappa \setminus \{0\}$$,
- $$q(\xi ):=\emptyset $$ for every $$\xi \in \kappa \setminus M$$.

Then *q* is a rectangular condition extending *p*, as sought. $\square$

## The failure of diamond on a Kurepa tree

The main result of this section is a consistent example of a binary Kurepa tree on which diamond fails. At the end of this section, we shall derive a few additional corollaries.

As a first step, we present a notion of forcing with side conditions for adding a branch through a given normal binary $\aleph _1$-tree *T*. The poset takes a transversal $\vec {t}$ of the complete binary tree  as a second parameter, and ensures that the generic branch will disagree on a club with this transversal.

### Definition 4.1

Let *T* be a normal binary $\aleph _1$-tree, and let .

The forcing $\mathbb {Q}(T,\,\vec {t})$ consists of all triples $p=(x_p,\mathcal {M}_p,f_p)$ that satisfy all of the following:

1. $x_p$ is a node in *T*;
2. $\mathcal {M}_p$ is a finite, $\in$-increasing chain of countable elementary submodels of $\operatorname {H}_{\omega _1}$;
3. $f_p$ is a partial function from $\mathcal {M}_p$ to $\omega _1$;
4. for every $$M\in \mathcal {M}_p$$:
  $${{\,\textrm{dom}\,}}(x_p)\ge M\cap \omega _1$$,
  $$x_p \mathbin \upharpoonright (M\cap \omega _1)\ne \vec {t}(M\cap \omega _1)$$;
  $$f_p\mathbin \upharpoonright M \in M$$.

The ordering is defined by letting $q \le p$ iff

1. $$x_q\supseteq x_p$$;
2. $$\mathcal {M}_q \supseteq \mathcal {M}_p$$;
3. for every $M \in {{\,\textrm{dom}\,}}(f_p)$, $M \in {{\,\textrm{dom}\,}}(f_q)$ and $f_q(M)\ge f_p(M)$.

### Remark 4.2

$$\mathbb {Q}(T,\,\vec {t})\subseteq \operatorname {H}_{\omega _1}$$, so that $$\mathbb {Q}(T,\,\vec {t})\in \operatorname {H}_{\mathfrak c^+}$$.

### Lemma 4.3

Suppose that *T* is a normal binary $\aleph _1$-Souslin tree, and . Let $p \in \mathbb {Q}(T,\,\vec {t})$. Then for every countable $M^*\prec \operatorname {H}_{\omega _2}$ with $\mathbb {Q}(T,\,\vec {t}) \in M^*$ such that $M^*\cap \operatorname {H}_{\omega _1}\in \mathcal {M}_p$, *p* is $M^*$-generic.

### Proof

Fix a model $M^*$ as above, and let $D \in M^*$ be a dense open subset of $\mathbb {Q}(T,\,\vec {t})$; we need to find an $r\in D\cap M^*$ compatible with *p*.

Fix a large enough $\gamma \in M^*\cap \omega _1$ such that $N\cap \omega _1\subseteq \gamma$ for every $N\in \mathcal {M}_p\cap M^*$. Define a condition

$$\begin{aligned} \bar{p}:=(x_p\mathbin \upharpoonright \gamma ,\mathcal {M}_p\cap M^*,f_p\mathbin \upharpoonright M^*), \end{aligned}$$

and note that $\bar{p} \in M^*$.

### Claim 4.3.1

The set $D':=\{x_q \mid q \in D,\; q \le \bar{p}\}$ is dense in $(T,{\supseteq })$ below $x_{\bar{p}}$.

### Proof

Let *y* be any extension of $x_{\bar{p}}$. Evidently, $(y,\mathcal {M}_{\bar{p}},f_{\bar{p}})$ is a legitimate condition. Pick $q \in D$ such that $q \le (y,\mathcal {M}_{\bar{p}},f_{\bar{p}})$. Then $x_q$ is an extension of *y* that lies in $D'$, as sought. $\square$

Denote $\delta :=M^*\cap \omega _1$. Since *T* is a Souslin tree lying in $M^*$, any node in $T_\delta$ is $(T,{\supseteq })$-generic over $M^*$. In particular, $x_p\mathbin \upharpoonright \delta$ is $(T,{\supseteq })$-generic over $M^*$, and it follows that there is an $x\in D'\cap M^*$ such that

$$\begin{aligned} x\subseteq x_p\mathbin \upharpoonright \delta . \end{aligned}$$

Now, pick any *q* witnessing $x \in D'$, so that $x=x_q\subseteq x_p$. By elementarity, we can assume that $q \in M^*$.

Define a condition *r* by letting $x_r:=x_p$, $\mathcal {M}_r:=\mathcal {M}_p\cup \mathcal {M}_q$, and $f_r$ be such that ${{\,\textrm{dom}\,}}(f_r)={{\,\textrm{dom}\,}}(f_p)\cup {{\,\textrm{dom}\,}}(f_q)$ and

$$\begin{aligned} f_r(\alpha ):=\max (\{f_p(\alpha )\mid \alpha \in {{\,\textrm{dom}\,}}(f_p)\}\cup \{f_q(\alpha )\mid \alpha \in {{\,\textrm{dom}\,}}(f_q)\}). \end{aligned}$$

It is not hard to see that *r* is a legitimate condition extending both *p* and *q*. $\square$

### Lemma 4.4

Suppose that *T* is a normal binary $\aleph _1$-Souslin tree, and . Then:

1. $$\mathbb {Q}(T,\,\vec {t})$$ is proper;
2. $\mathbb {Q}(T,\,\vec {t})$ adds a branch through *T* that evades $\vec {t}$ on a club.

### Proof

1. To verify properness, fix $p \in \mathbb {Q}(T,\,\vec {t})$, and a countable $M^* \prec \operatorname {H}_{\omega _2}$, containing everything relevant including *p*. Denote $\delta :=M^*\cap \omega _1$. As *T* is normal and Souslin, we may be able to extend $x_p$ to some node $x\in T_\delta$ distinct from $\vec {t}(\delta )$.
  The triple $(x,\mathcal {M}_p \cup \{M^*\cap \operatorname {H}_{\omega _1}\},f_p)$ is a condition in $\mathbb {Q}(T,\,\vec {t})$, and by Lemma 4.3, it is an $M^*$-generic condition.
2. Let *G* be a generic filter. It suffices to verify that the following uncountable set is moreover closed:
  $$\begin{aligned} D:=\{M\cap \omega _1 \mid \exists p \in G\,(M \in \mathcal {M}_p)\}, \end{aligned}$$
  that is, every $\gamma \in \kappa \setminus D$ is not an accumulation point of *D*. Let $\dot{D}$ be a name for the above set, and suppose $p \Vdash \dot{\gamma }\in \kappa \setminus \dot{D}$. By extending *p*, we can assume that it forces that $\dot{\gamma }=\gamma$, and $\gamma$ lies between two consecutive elements $N_0\in N_1$ of $\mathcal {M}_p$. By extending *p* further, we can assume that $f_p(N_0) \ge \gamma$. Since $\gamma$ is forced to not be the height of any element of $\mathcal {M}_p$, it follows that $N_0\cap \omega _1 < \gamma$, and
  $$\begin{aligned} p \Vdash \dot{D}\cap (N_0\cap \omega _1,\gamma ) =\emptyset , \end{aligned}$$
  as sought.

$$\square $$

### Definition 4.5

Given a sequence of binary $\aleph _1$-trees $T^0,\ldots ,T^{n}$, the tree product $T^0\otimes \cdots \otimes T^n$ is the collection of all $(x_0,\ldots ,x_n)\in T^0\times \cdots \times T^n$ such that ${{\,\textrm{dom}\,}}(x_0)=\cdots ={{\,\textrm{dom}\,}}(x_n)$, and the ordering is such that a node $(x_0,\ldots ,x_n)$ is below a node $(y_0,\ldots ,y_n)$ iff $x_i\subsetneq y_i$ for all $i\le n$.

In our context, it will be useful to know of the following fact which follows from [6, Corollary 5.7] as in the proof of [6, Corollary 5.3].

### Fact 4.6

([6, §5]) If $\diamondsuit ^+$ holds, then there is a binary $\aleph _1$-Aronszajn tree *T* and a sequence $\vec {T}=\langle T^\eta \mid \eta <\omega _2\rangle$ of normal binary $\aleph _1$-subtrees of *T* such that, for every nonempty $a\in [\omega _2]^{<\omega }$, the tree product $\bigotimes _{\eta \in a}T^\eta$ is an $\aleph _1$-Souslin tree.

It is well-known that if the product $T\otimes S$ of two normal binary $\aleph _1$-trees is Souslin, then

$$\begin{aligned} (T,{\supseteq }) \Vdash ``S \text { is Souslin}". \end{aligned}$$

Thus, it is natural to expect that furthermore

$$\begin{aligned} \mathbb {Q}(T,\,\vec {t}) \Vdash ``S \text { is Souslin}". \end{aligned}$$

The next lemma shows that this is indeed the case.

### Lemma 4.7

Suppose *T* and *S* are normal binary $\aleph _1$-trees the product of which is Souslin, and let . Then

$$\begin{aligned} \mathbb {Q}(T,\,\vec {t}) \Vdash ``S \text { is Souslin}". \end{aligned}$$

### Proof

Let $q \in \mathbb {Q}(T,\,\vec {t})$, and fix a $\mathbb {Q}(T,\,\vec {t})$-name $\dot{A}$ for a maximal antichain in *S*. Fix a countable $M^* \prec \operatorname {H}_{\omega _2}$ containing $\{\mathbb {Q}(T,\,\vec {t}), q, \dot{A}\}$. Denote $\delta := M^*\cap \omega _1$, and pick a node ${x}\in T_\delta \setminus \{\vec {t}(\delta )\}$ extending $x_q$. We define an extension $q'\le q$ by letting:

$$\begin{aligned} q':=(x,\mathcal {M}_q \cup \{M^*\cap \operatorname {H}_{\omega _1}\},f_q). \end{aligned}$$

It is clear that $q'$ is indeed a condition and $q' \le q$. We claim that

$$\begin{aligned} q' \Vdash ``\dot{A}\cap (S\mathbin \upharpoonright \delta )\text { is a maximal antichain in }S". \end{aligned}$$

To see this, pick a condition $p \le q'$ and a node ${y} \in S_\delta$; our aim is to find a condition $r\le p$ that forces *y* to extend an element of $\dot{A}$.

Fix a large enough $\gamma <\delta$ such that $N\cap \omega _1\subseteq \gamma$ for every $N\in \mathcal {M}_p\cap M^*$. Let $\bar{x}:=x_p\mathbin \upharpoonright \gamma$ and $\bar{y}:={y}\mathbin \upharpoonright \gamma$. Define a condition

$$\begin{aligned} \bar{p} := (\bar{x},\mathcal {M}_p\cap M^*,f_p \mathbin \upharpoonright M^*). \end{aligned}$$

Now, we turn to run a recursion producing a family

$$\begin{aligned} \mathcal {E}=\{(q_\alpha ,y_\alpha )\mid \alpha <\theta \}, \end{aligned}$$

satisfying that for each $\alpha$:

1. $$q_\alpha \in \mathbb {Q}(T,\,\vec {t})$$ with $$q_\alpha \le \bar{p}$$;
2. $$y_\alpha \in S$$ with $$\bar{y} \subseteq y_\alpha $$;
3. $$q_\alpha \Vdash \exists a \in \dot{A}\,(a \subseteq y_\alpha )$$;
4. $${{\,\textrm{dom}\,}}(x_{q_\alpha })={{\,\textrm{dom}\,}}(y_\alpha )$$;
5. for all $\beta <\alpha$, $(x_{q_\beta },y_\beta )\bot (x_{q_\alpha },y_\alpha )$ in $T\otimes S$.

We continue the construction until it is not possible to choose the next element. Because $T\otimes S$ is Souslin, Requirement (5) ensures that the construction terminates after countably many steps, so the ordinal $\theta$ ends up being countable.

### Claim 4.7.1

The set $\{(x_{q_\alpha },y_\alpha ) \mid \alpha <\theta \}$ is a maximal antichain above $(\bar{x},\bar{y})$ in $T \otimes S$.

### Proof

Suppose not. Then we may pick $(x',y')\in T \otimes S$ extending $(\bar{x},\bar{y})$ that is incompatible with $(x_{q_\alpha },y_\alpha )$ for every $\alpha <\theta$. By possibly extending both $x'$ and $y'$, we may assume that $x'=x_{q'}$ for some condition $q' \le \bar{p}$ which moreover satisfies:

$$\begin{aligned} q' \Vdash \exists a \in \dot{A}\,(a \subseteq y'). \end{aligned}$$

But now we can add $(q',y')$ to $\mathcal {E}$, contradicting its maximality. $\square$

Since $q \in M^*$, by making canonical choices in the recursive construction we may secure that the countable family $\mathcal {E}$ be a subset of $M^*$. So, by Claim 4.7.1, we may find an $\alpha <\theta$ such that ($x_{q_\alpha }\subseteq x$ and $y_\alpha \subseteq y$). Since $\mathcal {E}\subseteq M^*$, we infer that $q_\alpha \in M^*$. Now, as in the proof of Lemma 4.3, we see that there exists a condition *r* extending both *p* and $q_\alpha$. Recalling Requirement (3) in the choice of $q_\alpha$, we get that

$$\begin{aligned} r\Vdash \exists a \in \dot{A}\,(a \subseteq {y}), \end{aligned}$$

as sought. $$\square $$

We are now in conditions to prove the core of Theorem C.

### Theorem 4.8

It is consistent that there exists a binary Kurepa tree *T* such that $\diamondsuit (T)$ fails.

### Proof

We start with a model of $\textsf {GCH }$ and $\diamondsuit ^+$. Using Fact 4.6, fix a binary $\aleph _1$-tree *T* and a sequence $\vec {T}=\langle T^\xi \mid \xi <\omega _2\rangle$ of normal binary $\aleph _1$-subtrees of *T* such that $\bigotimes _{\xi \in a}T^\xi$ is an $\aleph _1$-Souslin tree for every nonempty $a\in [\omega _2]^{<\omega }$.^9^ Note that for all $\xi \ne \eta$, the trees $T^\xi$ and $T^\eta$ have a countable intersection.

Let $\mathcal {P}$ denote the collection of all pairs $(\mathbb {R},\tau )$ such that $\mathbb {R}$ is a notion of forcing lying in $\operatorname {H}_{\omega _2}$, and $\tau$ is a nice $\mathbb {R}$-name for an element of $\prod _{\alpha <\omega _1}T_\alpha$. Using $\textsf {GCH }$, we may fix a (repetitive) enumeration $\langle (\mathbb {R}_\xi ,\tau _\xi )\mid \xi <\omega _2\rangle$ of $\mathcal {P}$ in such a way that every pair is listed cofinally often.

Finally, we force with $\mathbb {P}_{\omega _2}$, where $(\langle \mathbb {P}_\xi \mid \xi \le \omega _2\rangle ,\langle \dot{\mathbb {Q}}_\xi \mid \xi <\omega _2\rangle )$ is the countable support iteration satisfying that for every $\xi <\omega _2$:

(i) $$\mathbb {P}_\xi \Vdash ``\dot{\mathbb {Q}}_\xi =\dot{\mathbb {Q}}(T^\xi ,\sigma _\xi )"$$, and
(ii) $\sigma _\xi$ is a nice $\mathbb {P}_\xi$-name for an element of $\prod _{\alpha <\omega _1}T_\alpha$ such that if $\mathbb {R}_\xi =\mathbb {P}_\eta$ for some $\eta \le \xi$, then $\sigma _\xi$ is the lift of $\tau _\xi$ (from a $\mathbb {P}_\eta$-name to a $\mathbb {P}_\xi$-name).

As $\textsf {CH }$ holds, and recalling Remark 4.2, we infer that $(\mathbb {P}_\xi ,\sigma _\xi )\in \mathcal {P}$ for all $\xi <\omega _2$.

### Claim 4.8.1

For every $\xi <\omega _2$, $\mathbb {P}_\xi$ forces that $\dot{\mathbb {Q}}(T^\xi ,\sigma _\xi )$ is proper.

### Proof

By Lemma 4.4(1), it suffices to prove that for every $\xi <\omega _2$,

$$\begin{aligned} \mathbb {P}_\xi \Vdash ``T^\xi \text { is Souslin}". \end{aligned}$$

We shall prove by induction on $\xi <\omega _2$ a stronger claim, namely that for every finite tuple $\xi<\eta _0<\ldots<\eta _n<\omega _2$,

$$\begin{aligned} \mathbb {P}_\xi \Vdash ``T^{\xi }\otimes T^{\eta _0}\otimes \cdots \otimes T^{\eta _n}\text { is Souslin}". \end{aligned}$$

There are three cases to consider:

- $\xi =0$. This is since the sequence $\vec {T}$ was given by Fact 4.6.
- $$\xi +1$$. Recall that
  $$\begin{aligned} \mathbb {P}_{\xi +1}\simeq \mathbb {P}_\xi *\dot{\mathbb {Q}}(T^\xi ,\sigma _\xi ). \end{aligned}$$
  Let $\xi +1<\eta _0<\ldots<\eta _n<\omega _2$ be a finite tuple. By the induction hypothesis,
  $$\begin{aligned} \mathbb {P}_\xi \Vdash ``T^\xi \otimes T^{\xi +1} \otimes T^{\eta _0} \otimes \cdots \otimes T^{\eta _n}\text { is Souslin}". \end{aligned}$$
  By invoking Lemma 4.7 in ${V}^{\mathbb {P}_\xi }$, we infer that
  $$\begin{aligned} \mathbb {P}_\xi *\dot{\mathbb {Q}}(T^\xi , \,\sigma _\xi ) \Vdash ``T^{\xi +1} \otimes T^{\eta _0} \otimes \cdots \otimes T^{\eta _n}\text { is Souslin}", \end{aligned}$$
  as sought.
- $\xi \in {{\,\textrm{acc}\,}}(\omega _2)$. We apply a result from [2, §3] or [28] stating that for any $\aleph _1$-tree *S*, the property “*S* *is Souslin*” is preserved at a limit stage of a countable support iteration of proper forcings.

$$\square $$

A standard name counting argument shows that $\textsf {CH }$ is preserved in every intermediate stage, so $\mathbb {P}_{\omega _2}$ satisfies the $\aleph _2$-cc.^10^ In addition, $\mathbb {P}_{\omega _2}$ is proper, so our forcing preserves all cardinals.

### Claim 4.8.2

$\mathbb {P}_{\omega _2}$ forces that *T* is Kurepa.

### Proof

For every $\xi <\omega _2$, $\mathbb {P}_{\omega _2}$ projects to a forcing of the form $\mathbb {Q}(T^\xi , \,\vec {t})$, and then Lemma 4.4(2) implies that in $V^{\mathbb {P}_{\omega _2}}$, there exists a branch $f_\xi$ through $T^\xi$, and in particular, through *T*. As the elements of $\langle T^\xi \mid \xi <\omega _2\rangle$ have a pairwise countable intersection, in $V^{\mathbb {P}_{\omega _2}}$, $\langle f_\xi \mid \xi <\omega _2\rangle$ is injective. $\square$

### Claim 4.8.3

$\mathbb {P}_{\omega _2}$ forces that $\diamondsuit (T)$ fails.

### Proof

Otherwise, as $\prod _{\alpha <\omega _1}T_\alpha$ lies in the ground model, we may fix a nice $\mathbb {P}_{\omega _2}$-name $\dot{t}$ for a transversal $\vec {t}\in \prod _{\alpha <\omega _1}T_\alpha$ that witnesses $\diamondsuit (T)$ in the extension. As $\mathbb {P}_{\omega _2}$ has the $\aleph _2$-cc and $\dot{t}$ is a nice name for an $\aleph _1$-sized set, there is a large enough $\eta <\omega _2$ such that all nontrivial conditions appearing in $\dot{t}$ belong to $\mathbb {P}_\eta$. It thus follows that $\vec {t}$ admits a nice $\mathbb {P}_\eta$-name, say, $\tau$. Clearly, $(\mathbb {P}_\eta ,\tau )\in \mathcal {P}$, so we may find a large enough $\xi \in [\eta ,\omega _2)$ such that $(\mathbb {R}_\xi ,\tau _\xi )=(\mathbb {P}_\eta ,\tau )$. Recalling Clauses (i) and (ii) in the definition of our iteration, it is the case that $\mathbb {P}_{\xi +1}\simeq \mathbb {P}_\xi *\dot{\mathbb {Q}}(T^\xi , \sigma _\xi )$ where $\sigma _\xi$ is a $\mathbb {P}_\xi$-name for $\vec {t}$. By Lemma 4.4(2), then, $\mathbb {P}_{\xi +1}$ introduces a branch through *T* that evades $\vec {t}$ on a club. So $\vec {t}$ cannot witness $\diamondsuit (T)$ in $\mathbb {P}_{\omega _2}$. This is a contradiction. $\square$

This completes the proof. $\square$

### Ramifications

Proposition 2.12 may suggest that the validity of $\diamondsuit (T)$ for a Kurepa tree *T* depends only on the cardinal $|\mathcal {B}(T)|$. However, the next corollary shows that this is not the case:

#### Corollary 4.9

It is consistent that there exist two binary Kurepa trees $T'$ and *T* such that $|\mathcal {B}(T')|=|\mathcal {B}(T)|=2^{\aleph _1}$, $\diamondsuit (T')$ holds, but $\diamondsuit (T)$ fails.

#### Proof

Work in $\mathsf L$. By Corollary 3.4, we may fix a binary Kurepa tree $T'$ such that $\diamondsuit (T')$ holds in any forcing extension preserving $\omega _1$, $\omega _2$, and the stationary subsets of $\omega _1$. As $\mathsf L$ satisfies $\textsf {GCH }$ and $\diamondsuit ^+$, the proof of Theorem 4.8 provides us with an $\aleph _2$-cc proper notion of forcing $\mathbb {P}_{\omega _2}$ that introduces a binary Kurepa tree *T* on which $\diamondsuit$ fails. Altogether, $\mathsf L^{\mathbb {P}_{\omega _2}}$ is a model satisfying the desired configuration. $\square$

#### Corollary 4.10

It is consistent that there exists a binary Kurepa tree *T* such that $\diamondsuit (T)$ fails and *T* is uniformly homogeneous.^11^

#### Proof

The proof is almost identical to that of Theorem 4.8. We start with a model of $\textsf {GCH }$ and $\diamondsuit ^+$. Using Fact 4.6, we fix a binary $\aleph _1$-tree *T* and a sequence $\langle T^\xi \mid \xi <\omega _2\rangle$ of normal binary $\aleph _1$-subtrees of *T* such that $\bigotimes _{\xi \in a}T^\xi$ is an $\aleph _1$-Souslin tree for every nonempty $a\in [\omega _2]^{<\omega }$. Now, let $T'$ be the collection of all functions  for which there exists some $t\in T$ such that ${{\,\textrm{dom}\,}}(t')={{\,\textrm{dom}\,}}(t)$ and $\{ \alpha \in {{\,\textrm{dom}\,}}(t)\mid t(\alpha )\ne t'(\alpha )\}$ is finite. Then $T'$ is a uniformly homogeneous $\aleph _1$-tree having each of the $T^\xi$’s as a normal binary $\aleph _1$-subtree. So, we can continue with the proof of Theorem 4.8 using $T'$ instead of *T*. $\square$

#### Corollary 4.11

It is consistent that there exists a binary Kurepa tree *S* such that $\diamondsuit (S)$ fails and *S* is rigid.

#### Proof

The proof is quite close to that of Theorem 4.8. We start with a model of $\textsf {GCH }$ and $\diamondsuit ^+$. In particular, there exists a binary Kurepa tree. Setting $(\kappa ,\mho ):=(\aleph _1,\aleph _2)$, this means that there exists a $\kappa$-tree with $\mho$-many cofinal branches. Instead of using Fact 4.6, we appeal to [6, Corollary 5.7] with $\kappa , \mho$ and $\varsigma :=\omega$ to obtain a downward-closed subfamily  such that $(T,{\subsetneq })$ is an Aronszajn tree, every node in *T* admits infinitely many immediate successors, and there exists a sequence $\langle T^\xi \mid \xi <\omega _2\rangle$ of normal downward-closed subtrees of *T* such that $\bigotimes _{\xi \in a}T^\xi$ is an $\aleph _1$-Souslin tree for every nonempty $a\in [\omega _2]^{<\omega }$. We force with $\mathbb {P}_{\omega _2}$, where $(\langle \mathbb {P}_\xi \mid \xi \le \omega _2\rangle ,\langle \dot{\mathbb {Q}}_\xi \mid \xi <\omega _2\rangle )$ is the countable support iteration satisfying that for every $\xi <\omega _2$, $\mathbb {P}_\xi \Vdash ``\dot{\mathbb {Q}}_\xi =\dot{\mathbb {Q}}(T^\xi ,\sigma _\xi )"$, where $\sigma _\xi$ is a nice $\mathbb {P}_\xi$-name for an element of $\prod _{\alpha <\omega _1}T_\alpha$ obtained from some bookkeeping sequence. This time, a typical transversal  instead of , but everything goes through and we end up in a generic extension in which *T* is a Kurepa tree on which diamond fails.

Next, let *S* be the binary $\aleph _1$-tree produced by the proof of Lemma 2.1 when fed with the tree $\textbf{T}:=(T,{\subsetneq })$. Since $\textbf{T}$ is Kurepa on which diamond fails, *S* is a binary Kurepa tree and $\diamondsuit (S)$ fails.

Recall that the proof of Lemma 2.1 made use of an injective sequence $\langle r_m\mid m<\omega \rangle$ of functions from $\omega$ to 2. For our purpose here, we shall moreover assume that for all $m\ne m'$, it is the case that $\Delta (r_m,r_{m'})=\min \{m,m'\}$.^12^

To prove that *S* is rigid, we must establish the following.

#### Claim 4.11.1

Suppose that $\pi :S\leftrightarrow S$ is an automorphism of $(S,{\subsetneq })$. Then $\pi$ is the identity map.

#### Proof

Suppose not, so that $\pi (s)\ne s$ for some $s\in S$. By the definition of *S* and as $\pi$ is order-preserving, it follows that there exists a $t\in T$ such that $\pi (\psi (t))\ne \psi (t)$. Let $\beta <\omega _1$ be the least for which there exists a $t\in T_\beta$ with $\pi (\psi (t))\ne \psi (t)$. Clearly, $\beta$ is a successor ordinal, say, $\beta =\alpha +1$. Fix $t_0\ne t_1$ in $T_{\alpha +1}$ such that $\pi (\psi (t_0))=\psi (t_1)$ and note that the minimality of $\beta$ implies that $t_0\mathbin \upharpoonright \alpha =t_1\mathbin \upharpoonright \alpha$, which we hereafter denote by *t*. Now, since every node in *T* admits infinitely many immediate successors, we may fix $t_2,t_3\in T_{\alpha +1}$ such that:

- $t_2,t_3$ are immediate successors of *t*,^13^
- $$\pi (\psi (t_2))=\psi (t_3)$$, and
- $$\min \{\varphi _\alpha (t_2),\varphi _\alpha (t_3)\}>\max \{\varphi _\alpha (t_0),\varphi _\alpha (t_1)\}$$.

Recalling the proof of Lemma 2.1, for every $i<4$, letting $m_i:=\varphi _\alpha (t_i)$, it is the case thatAs $m_0<m_2$, $\Delta (r_{m_0},r_{m_2})=m_0$ and

$$\begin{aligned} \Delta (\psi (t_0),\psi (t_2))={\omega \cdot \alpha }\mathrel {+}m_0. \end{aligned}$$

Likewise,

$$\begin{aligned} \Delta (\pi (\psi (t_0)),\pi (\psi (t_2)))=\Delta (\psi (t_1),\psi (t_3))={\omega \cdot \alpha }\mathrel {+}m_1. \end{aligned}$$

As $m_0\ne m_1$, we infer that

$$\begin{aligned} \Delta (\psi (t_0),\psi (t_2))\ne \Delta (\pi (\psi (t_0)),\pi (\psi (t_2))), \end{aligned}$$

contradicting the fact that $\pi$ is an automorphism of *S*. $\square$

This completes the proof. $\square$

## Data Availability

Data sharing not applicable to this article as datasets were neither generated nor analyzed.

## Footnotes

1 It follows that we may assume that $\langle t_\alpha \mid \alpha <\omega _1\rangle$ is a transversal for $\prod _{\alpha <\omega _1}\{t\in T_\alpha \mid t\text { is extended }\text {by some element of }\mathcal {B}(T)\}$.

2 These trees were first considered by Mitchell [27], and the naming comes from [42]. Baumgartner and Davies [4, 7] call them *Canadian trees*.

3 For every regular uncountable $\kappa =\kappa ^{<\kappa }$, Martin’s axiom together with $2^{\aleph _0}=\kappa$ may be introduced by a ccc (in particular, proper) forcing (see [24, Theorem VIII.6.3]).

4 A proper forcing may kill the Kurepa-ness of *T*, but then $\diamondsuit (T)$ will hold trivially.

5 For a set of ordinals *A*, we write ${{\,\textrm{acc}\,}}(A):=\{\alpha \in A\mid \sup (\alpha )=\alpha >0\}$.

6 The injectivity here follows from Hausdorff-ness.

7 That is, if the real line cannot be covered by $|\mathcal {B}(\textbf{T})|$-many meager sets.

8 This was generalized by Hayut and Müller [16, Lemma 15] to any successor of a regular uncountable cardinal $\kappa$ such that $\kappa ^+=(\kappa ^+)^{\textsf{L}}$.

9 The tree *T* given by the fact is moreover Aronszajn, but this feature is not necessary for our application here.

10 Recall [4, Theorem 2.2].

11 The definition of a *uniformly homogeneous* tree may be found in [6, §4].

12 This can easily be arranged by defining $r_m:\omega \rightarrow 2$ via $r_m(n):=1$ iff $n<m$.

13 Possibly, $$t_2=t_3$$.

## References

[1] Abraham, U.: Proper forcing. In: Handbook of Set Theory. Vol. 1, 2, 3, pp. 333–394. Springer, Dordrecht (2010)

[2] Abraham, U., Shelah, S.: A well-order of the reals and incompactness of . Ann. Pure Appl. Logic **59**, 1–32 (1993)

[3] Bartoszyński, T., Judah, H.: Set Theory. A K Peters, Ltd., Wellesley, MA (1995). On the structure of the real line

[4] Baumgartner, J.E.: Iterated forcing. In: Surveys in set theory, volume 87 of London Math. Soc. Lecture Note Ser., pp. 1–59. Cambridge Univ. Press, Cambridge (1983)

[5] Baumgartner, J.E.: Applications of the proper forcing axiom. In: Handbook of set-theoretic topology, pp. 913–959. North-Holland, Amsterdam (1984)

[6] Brodsky, A.M., Rinot, A., Yadai, S.: Proxy principles in combinatorial set theory. In: Selected Topics in Combinatorial Analysis, Part II, vol. 22(30), pp. 89–136. Mathematical Institute of the Serbian Academy of Sciences and Arts (2025)

[7] Davies, P.C.: Small Baire spaces and sigma-dense partial orders. ProQuest LLC, Ann Arbor, MI (1979). Thesis (Ph.D.)–University of Toronto (Canada)

[8] Devlin, K.J.: A note on the combinatorial principles . Proc. Amer. Math. Soc. **72**(1), 163–165 (1978)

[9] Devlin, K.J.: Variations on . J. Symbolic Logic **44**(1), 51–58 (1979)

[10] Devlin, K.J., Johnsbråten, H.: The Souslin problem. Lecture Notes in Mathematics, vol. 405. Springer-Verlag, Berlin-New York (1974)

[11] Devlin, K.J., Shelah, S.: A weak version of which follows from . Israel J. Math. **29**(2–3), 239–247 (1978)

[12] Fuchino, S., Shelah, S., Soukup, L.: Sticks and clubs. Ann. Pure Appl. Logic **90**(1–3), 57–77 (1997)

[13] Giron, I., Hayut, Y.: Sealed Kurepa trees (2023). arXiv:2401.00572

[14] Gitik, M., Rinot, A.: The failure of diamond on a reflecting stationary set. Trans. Amer. Math. Soc. **364**(4), 1771–1795 (2012)

[15] Gödel, K.: The Consistency of the Continuum Hypothesis. Annals of Mathematics Studies, no. 3. Princeton University Press, Princeton, N. J. (1940)

[16] Hayut, Y., Müller, S.: Perfect subtree property for weakly compact cardinals. Israel J. Math. **253**(2), 865–886 (2023)

[17] Higman, G., Stone, A.H.: On inverse systems with trivial limits. J. London Math. Soc. **29**, 233–236 (1954)

[18] Inamdar, T., Rinot, A.: Was Ulam right? II: small width and general ideals. Algebra Universalis **85**(2), 14 (2024)

[19] Jech, T.J.: Trees. J. Symb. Log. **36**(1), 1–14 (1971)

[20] Jensen, R.B.: The fine structure of the constructible hierarchy. Ann. Math. Logic **4**, 229–308 (1972)

[21] Jensen, R.B.: Souslin’s hypothesis is incompatible with V = L. Notices Amer. Math. Soc **15**(6), 935 (1968)

[22] Jensen, R.B., Kunen, K.: Some combinatorial properties of l and v, handwritten notes, (1969)

[23] Koszmider, P.: Kurepa trees and topological non-reflection. Topology Appl. **151**(1–3), 77–98 (2005)

[24] Kunen, K.: Set theory, volume 102 of Studies in Logic and the Foundations of Mathematics. North-Holland Publishing Co., Amsterdam (1980). An introduction to independence proofs

[25] Kurepa, D.: Ensembles ordonnés et ramifiés. Publications de l’Institut Mathématique Beograd **4**, 1–138 (1935)

[26] Matet, P.: Corrigendum to: Partitions and diamond [Proc. Amer. Math. Soc. **97** (1986), no. 1, 133–135; MR0831401 (87e:03111)]. *Proc. Amer. Math. Soc.*, 101(3), 519–520 (1987)

[27] Mitchell, W.: Aronszajn trees and the independence of the transfer property. Ann. Math. Logic, **5** 21–46 (1972/73)

[28] Miyamoto, T.: -Souslin trees under countable support iterations. Fund. Math. **142**(3), 257–261 (1993)

[29] Moore, J.T., Hrušák, M., Džamonja, M.: Parametrized principles. Trans. Amer. Math. Soc. **356**(6), 2281–2306 (2004)

[30] Poór, M., Shelah, S.: Characterizing the spectra of cardinalities of branches of kurepa trees. Pac. J. Math. **311**(2), 423–453 (2021)

[31] Sakai, H.: Ideals over and combinatorical principles on . http://www2.kobe-u.ac.jp/~hsakai/Research/works.html (2006). Unpublished note from 2/October/2006

[32] Shelah, S.: Infinite abelian groups, Whitehead problem and some constructions. Israel J. Math. **18**, 243–256 (1974)

[33] Shelah, S.: Proper and improper forcing, 2nd edn. Perspectives in Mathematical Logic. Springer-Verlag, Berlin (1998)

[34] Shelah, S.: Middle diamond. Arch. Math. Logic **44**, 527–560 (2005)

[35] Shelah, S.: Diamonds. Proc. Amer. Math. Soc. **138**(6), 2151–2161 (2010)

[36] Silver, J.: The independence of Kurepa’s conjecture and two-cardinal conjectures in model theory. In: Axiomatic Set Theory (Proc. Sympos. Pure Math., Vol. XIII, Part I, Univ. California, Los Angeles, Calif., 1967), pp. 383–390. Amer. Math. Soc., Providence, R.I. (1971)

[37] Solovay, R.M., Tennenbaum, S.: Iterated Cohen extensions and Souslin’s problem. Ann. Math. **2**(94), 201–245 (1971)

[38] Souslin, M.Y.: Problème 3. Fundam. Math. **1**(1), 223 (1920)

[39] Steinhorn, C.I., King, J.H.: The uniformization property for . Israel J. Math. **36**(3–4), 248–256 (1980)

[40] Stewart, D.H.: The consistency of the kurepa hypothesis with the axioms of set theory. Master’s thesis, University of Bristol (1966)

[41] Todorčević, S.: Trees and linearly ordered sets. In: Handbook of Set-Theoretic Topology, pp. 235–293. North-Holland, Amsterdam (1984)

[42] Todorčević, S.B.: Some consequences of . Topology Appl. **12**(2), 187–202 (1981)

[43] Ulam, S.M.: Zur Masstheorie in der allgemeinen Mengenlehre. Uniwersytet, seminarjum matematyczne (1930)

[44] Unger, S.: Aronszajn trees and the successors of a singular cardinal. Arch. Math. Logic **52**(5–6), 483–496 (2013)

